# The Features of Fecal and Ileal Mucosa-Associated Microbiota in Dairy Calves during Early Infection with *Mycobacterium avium* Subspecies *paratuberculosis*

**DOI:** 10.3389/fmicb.2016.00426

**Published:** 2016-03-31

**Authors:** Hooman Derakhshani, Jeroen De Buck, Rienske Mortier, Herman W. Barkema, Denis O. Krause, Ehsan Khafipour

**Affiliations:** ^1^Department of Animal Science, University of ManitobaWinnipeg, MB, Canada; ^2^Department of Production Animal Health, University of CalgaryCalgary, AB, Canada; ^3^Department of Medical Microbiology, University of ManitobaWinnipeg, MB, Canada

**Keywords:** *Mycobacterium avium* subspecies *paratuberculosis* (MAP), Johne's disease (JD), microbiota, dysbiosis, gut inflammation

## Abstract

Current diagnostic tests for Johne's disease (JD), a chronic granulomatous inflammation of the gastrointestinal tract of ruminants caused by *Mycobacterium avium* subspecies *paratuberculosis* (MAP), lack the sensitivity to identify infected animals at early (asymptomatic) stages of the disease. The objective was to determine the pattern of MAP-associated dysbiosis of intestinal microbiota as a potential biomarker for early detection of infected cattle. To that end, genomic DNA was extracted from ileal mucosa and fecal samples collected from 28 MAP-positive and five control calves. High-throughput Illumina sequencing of the V4 hypervariable region of the 16S rRNA gene was used for community profiling of ileal mucosa-associated (MAM) or fecal microbiota. The PERMANOVA analysis of unweighted UniFrac distances revealed distinct clustering of ileal MAM (*P* = 0.049) and fecal microbiota (*P* = 0.068) in MAP-infected vs. control cattle. Microbiota profile of MAP-infected animals was further investigated by linear discriminant analysis effective size (LEfSe); several bacterial taxa within the phylum Proteobacteria were overrepresented in ileal MAM of control calves. Moreover, based on reconstructed metagenomes (PICRUSt) of ileal MAM, functional pathways associated with MAP infection were inferred. Enrichment of lysine and histidine metabolism pathways, and underrepresentation of glutathione metabolism and leucine and isoleucine degradation pathways in MAP-infected calves suggested potential contributions of ileal MAM in development of intestinal inflammation. Finally, simultaneous overrepresentation of families Planococcaceae and Paraprevotellaceae, as well as underrepresentation of genera *Faecalibacterium* and *Akkermansia* in the fecal microbiota of infected cattle, served as potential biomarker for identifying infected cattle during subclinical stages of JD. Collectively, based on compositional and functional shifts in intestinal microbiota of infected cattle, we inferred that this dynamic network of microorganisms had an active role in intestinal homeostasis.

## Introduction

*Mycobacterium avium* subspecies *paratuberculosis* (MAP) is the causative agent of Johne's disease (JD), a chronic granulomatous inflammation of the gastrointestinal (GI) tract in ruminants. Numerous US and Canadian dairy herds are infected with MAP with a prevalence ranging from 21 to 93% depending on region and testing methods used to identify infected herds (Losinger, 2005; Tiwari et al., 2006; Pillars et al., 2009). While the high prevalence of MAP infection has imposed substantial economic losses to the North American dairy and beef industries (~$79/cow/year for infected dairy herds; Losinger, 2005; Tiwari et al., 2006; Pillars et al., 2009), it is also a public health threat since several studies have indicated a potential association between MAP and Crohn's disease (CD) in humans (Scanu et al., 2007; Mendoza et al., 2010).

Fecal–oral transmission is the primary mechanism of MAP infection, with newborn calves apparently being most susceptible (Windsor and Whittington, 2010). This initial infection is usually followed by a prolonged (>2 years) incubation period (Over et al., 2011), after which cattle exhibit clinical signs. Several methods for detection of MAP have been developed, including fecal culture and PCR, and serum/milk ELISA for detection of MAP-specific antibodies. Although, sensitivity of these methods is high for detection of infected cattle in later stages of disease, sensitivity to detect MAP infection during its early subclinical stages is low (Tiwari et al., 2006; Sorge et al., 2011). Therefore, there is an immediate need to identify novel approaches for early diagnosis of MAP in cattle. Recent advances in proteomics, transcriptomics, and metabolomics have prompted a search for potential biomarkers of CD (Jansson et al., 2009; Erickson et al., 2012; Faubion et al., 2013), tuberculosis (Walzl et al., 2011), and MAP (You et al., 2012; David et al., 2014; De Buck et al., 2014). In addition, next-generation sequencing technologies and metagenomics also have potential applications in biomarker discovery. The composition of gut microbiota, a complex network of microorganisms within virtually all vertebrates (Ley et al., 2008), can be readily determined by variations in bacterial 16S rRNA gene sequences (Hooper et al., 2012). Furthermore, inflammatory responses in the GI tract can disturb the normal habitant of the resident microbiota and alter its composition and functional properties (dysbiosis; Collins and Bercik, 2009); it is noteworthy that the profile of these changes has been extensively used for biomarker discovery for inflammatory bowel disease (IBD) in humans (Berry and Reinisch, 2013).

It is well-established that proliferation of MAP in the ileal mucosa and regional lymph nodes incites several cellular and humoral immune responses (Over et al., 2011). Following ingestion, MAP undergoes endocytosis by M cells of Peyer's patches and subsequently phagocytosis by macrophages, where the bacteria resist intracellular degradation (by unknown mechanisms), thereby inhibiting maturation of phagosomes (Momotani et al., 1988; Weiss et al., 2006). Alternate immune responses are then launched through a complex network of cytokines and receptors, including CD4^+^ T cells and cytolytic CD8^+^ T cells, which eventually lead to secretion of proinflammatory cytokines, e.g., interferon-γ, tumor necrosis factor alpha (TNF-α) and interleukin (IL) 2 (Coussens, 2001). Consequently, microscopic and eventually macroscopic lesions usually develop in the intestinal epithelium 1–3 months after infection (Payne and Rankin, 1961; Buergelt et al., 1978). However, there is a paucity of knowledge regarding impacts of the aforementioned inflammatory responses on composition and functional properties of intestinal microbiota. Therefore, we hypothesized that due to modulatory interactions between the host immune system and intestinal microbiota, microbial dysbiosis occurs during subclinical stages of JD. Objectives were to: (1) characterize the profile of MAP-associated microbial dysbiosis in the ileal mucosa-associated microbiota (MAM) and the fecal samples; and (2) determine whether the pattern of fecal microbial dysbiosis was appropriate for detection of subclinical JD. To those ends, dairy calves at various ages were experimentally inoculated with either high or low doses of MAP and infection subsequently confirmed by tissue culture. High-throughput Illumina sequencing of the bacterial 16S rRNA genes was used to characterize composition of microbial communities and predict their functional properties (MAP-infected and control calves).

## Materials and methods

### Animal experiment

**Figure 1:**
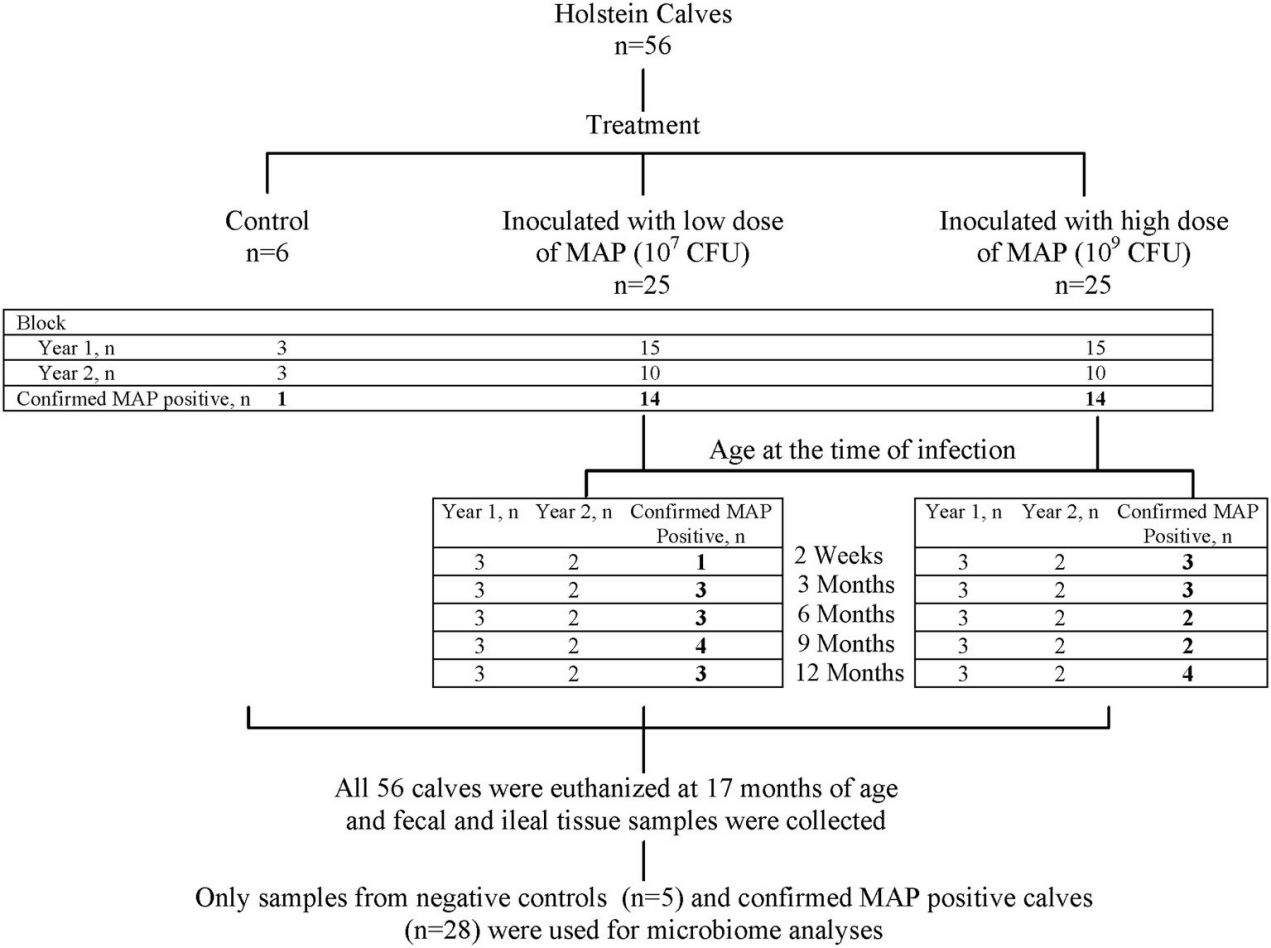
**Summary of study design**. The experiment was conducted in two replicates (consecutive experimental years) with first 30 and then 20 inoculated calves equally representing all age and dose groups. Overall, 28 out of 50 inoculated calves were identified (indicated by bold numbers) as MAP-positive (based on detection of at least one tissue culture positive, the “gold standard” for identification of MAP infection; Mortier et al., 2013), and along with five control calves were selected for 16S rRNA profiling of the ileal mucosa-associated (MAM) and fecal micoribota.

Details of the animal experiment and sampling procedures have been reported (Mortier et al., 2013). In brief, Holstein–Friesian bull calves (*n* = 56) were purchased from MAP-free or low prevalence dairy farms across Alberta, Canada. Inclusion criteria were based on negative pooled (*n* = 5) fecal cultures (decontaminated and prepared for culture according to manufacturer's instructions; para-JEM®, TREK Diagnostic systems, Oakwood Village, OH, USA), and a within-herd seroprevalence < 5% (IDEXX Paratuberculosis Ab Test; IDEXX Laboratories Inc., Westbrook, ME, USA) to minimize the risk of including calves that had acquired intra-uterine MAP infection. Calves were born on-farm, in the presence of a member of the research team, to prevent contact with the dam or environment, and then transferred to a biosecurity level 2 housing facility with individual custom-built housing units. A virulent cattle type MAP strain isolated from a clinical case, with an identical IS900—RFLP profile as the reference strain K10 (data not shown)—the recommended strain type to use in experimental infections (Hines et al., 2007)—was grown in supplemented 7H9 broth and used as inoculum. High dose (HD, 5 × 10^9^ CFU) and low dose (LD, 5 × 10^7^ CFU) inoculums were prepared to be given on 2 consecutive days, corresponding to five times the recommended standard bovine challenge dose (Hines et al., 2007) and 10 times the lowest confirmed and consistent infectious dose for young calves (Sweeney et al., 2006), respectively. The 56 calves were randomly allocated to 5 age groups (2 weeks, and 3, 6, 9, and 12 months of age at the time of inoculation; *n* = 10 calves/age group × 5 age group = 50) receiving either HD (*n* = 5 calves/dose group/age group × 5 age group = 25) or LD (*n* = 25), and a control group (*n* = 6). The experiment was conducted in two blocks (2 consecutive experimental years) with the first 30 and then 20 inoculated calves equally representing all age and dose groups, with three controls included in each block (Figure 1). Animal care protocols M09083 and M09050 were approved by the University of Calgary Animal Care Committee.

### Necropsy, tissue culture, and selection of animals for microbiome study

Following euthanasia (Euthanyl Forte®, Bimeda-MTC Animal Health Inc., Cambridge, ON, Canada) at 17 months of age, necropsies were performed immediately on 21 tissue sites, as described (Mortier et al., 2013). Macroscopic lesions were assessed as: 0 = no macroscopic changes; 1 = 1 enlarged or edematous lymph node (LN) of the small intestine or liver; 2 = multiple enlarged and edematous mesenteric LN and/or hyperemia of the ileocaecal valve; 3 = enlarged mesenteric LN and/or mild to moderate thickening of ileal or jejunal mucosa; and 4 = enlarged mesenteric LN and severe thickening and corrugation of the ileal, jejunal, and colon mucosa. Furthermore, samples from the ileocaecal valve, ileocaecal LN, ileal LN and distal ileum were subjected to histopathological examinations to assess paratuberculosis-associated histological lesions as follows: 0 = no lesions; 1 = focal lesions; 2 = multifocal lesions; and 3 = diffuse lymphocytic, multibacillary, or intermediate lesions.

From each of the 21 tissue sampling sites, a 2-g sample was processed and cultured for identification of MAP-positive calves, as described (Mortier et al., 2013). Tissue culture was considered the gold standard in detection of MAP infection (Tiwari et al., 2006); therefore, selection of MAP-inoculated calves to be included in the current study was based on detection of at least one tissue-positive site in that calf. Furthermore, control calves identified as MAP-positive (based on tissue culture) were excluded from downstream microbiome analysis.

### Sample processing, DNA extraction, and quality check

Genomic DNA was extracted from ileal MAM and fecal microbiota. Frozen distal-ileum tissue samples were thawed at 4°C overnight. Tissue samples were then rinsed carefully with sterile PBS and the mucosa associated-layer was obtained by scraping with a sterile scalpel blade. For each sample, 100 mg was used for extraction of bacterial genomic DNA using ZR-96 Tissue and Insect DNA Kit (Zymo Research, Irvine, CA, USA). Fecal samples were collected from the distal colon; 30 g of each sample was carefully homogenized cryogenically using Geno/Grinder® 2010 (SPEX SamplePrep, Metuchen, NJ, USA) and DNA extracted using a ZR-96 Fecal DNAKit (Zymo Research, Irvine, CA, USA). Both extraction kits included a bead-beating step using the ZR BashingBead racks (Zymo Research, Irvine, CA, USA) for mechanical lysis of the cells. Thereafter, DNA was quantified using a NanoDrop 2000 spectrophotometer (Thermo Scientific, Waltham, MA, USA). The DNA samples were normalized to 20 ng/μl, and quality verified by PCR amplification of the 16S rRNA gene using universal primers 27F (5′-GAAGAGTTTGATCATGGCTCAG-3′) and 342R (5′-CTGCTGCCTCCCGTAG-3′), as described (Khafipour et al., 2009). Amplicons were verified by agarose gel electrophoresis.

### Library construction and illumina sequencing

The PCR amplification was targeted to amplify the V4 region of 16S rRNA gene using modified F515/R806 primers (Caporaso et al., 2012) as described (Derakhshani et al., 2016). The reverse PCR primer was indexed with 12-base Golay barcodes, allowing for multiplexing of samples. For each sample, the PCR reaction was performed in duplicate and contained 1.0 μl of pre-normalized DNA, 1.0 μl of each forward and reverse primer (10 μM), 12 μl HPLC grade water (Fisher Scientific, Ottawa, ON, Canada), and 10 μl of 5 Prime Hot MasterMix (5 Prime Inc., Gaithersburg, MD, USA). Reactions consisted of an initial denaturing step at 94°C for 3 min followed by 35 amplification cycles at 94°C for 45 s, 50°C for 60 s, and 72°C for 90 s, with a final extension step at 72°C for 10 min in an Eppendorf Mastercycler pro (Eppendorf, Hamburg, Germany). Then, PCR products were purified using a ZR-96 DNA Clean-up Kit (ZYMO Research, Irvine, CA, USA) to remove primers, dNTPs, and reaction components. The V4 library was then generated by pooling 200 ng of each sample, quantified by Picogreen dsDNA (Invitrogen, Burlington, ON, Canada). This was followed by multiple dilution steps using pre-chilled hybridization buffer (HT1; Illumina, San Diego, CA, USA) to bring the pooled amplicons to a final concentration of 5 pM, as determined with a Qubit 2.0 Fluorometer (Life Technologies, Burlington, ON, Canada). Finally, 15% of the PhiX control library was spiked into the amplicon pool to improve the unbalanced and biased base composition, a common characteristic of low-diversity 16S rRNA libraries. Customized sequencing primers for read1 (5′-TATGGTAATTGTGTGCCAGCMGCCGCGGTAA-3′), read2 (5′- AGTCAGTCAGCCGGACTACHVGGGTWTCTA AT-3′), and index read (5′-ATTAGAWACCCBDGTAGTCC GGCTGACTGACT-3′) were synthesized and purified by polyacrylamide gel electrophoresis (Integrated DNA Technologies, Coralville, IA, USA) and added to the MiSeq Reagent Kit v2 (300-cycle; Illumina, San Diego, CA, USA). The 150 paired-end sequencing reaction was performed on a MiSeq platform (Illumina) at the Gut Microbiome and Large Animal Biosecurity Laboratories, Department of Animal Science, University of Manitoba, Winnipeg, MB, Canada. The sequencing data were deposited into the Sequence Read Archive (SRA) of NCBI (http://www.ncbi.nlm.nih.gov/sra) and can be accessed via accession number SRR2181770.

### Bioinformatic analyses

The PANDAseq assembler (Masella et al., 2012) was used to merge and fix overlapping paired-end Illumina fastq files. All sequences with mismatches or ambiguous calls in the overlapping region were discarded. The output fastq file was analyzed by downstream computational pipelines of the open source software package QIIME (Caporaso et al., 2010b). Assembled reads were de-multiplexed according to barcode sequences; those with a read length < 175 bases were removed from downstream analysis. Chimeric reads were filtered using UCHIME (Edgar et al., 2011) and sequences were assigned to Operational Taxonomic Units (OTU) using the QIIME implementation of UCLUST (Edgar, 2010) at 97% pairwise identity threshold. Taxonomies were assigned to the representative sequence of each OTU using RDP classifier (Wang et al., 2007) and aligned with the Greengenes Core reference database (DeSantis et al., 2006) using PyNAST algorithms (Caporaso et al., 2010a). A phylogenetic tree was built with FastTree 2.1.3. (Price et al., 2010) for further comparisons between microbial communities.

**Figure 2:**
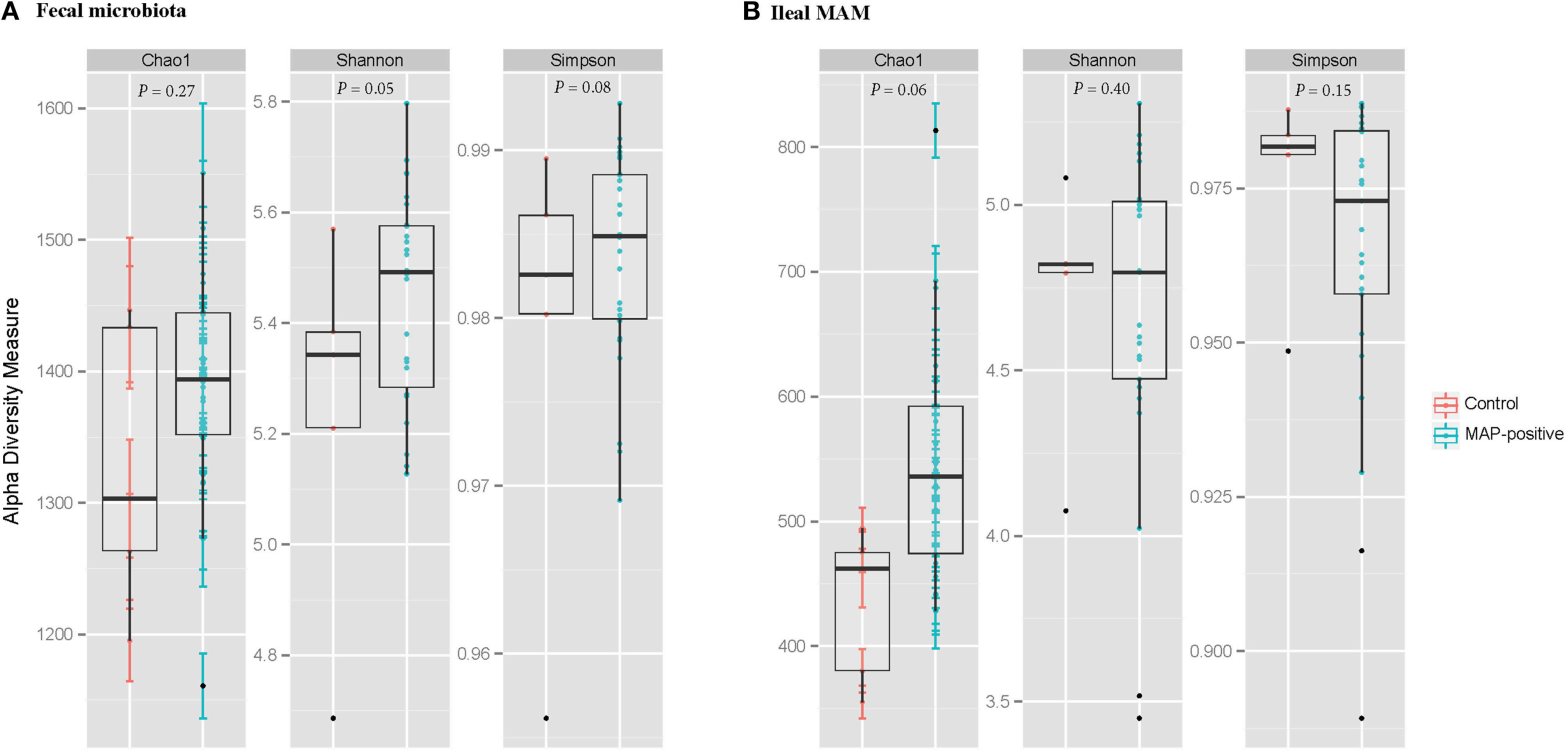
**Graphic summary of alpha-diversity indices**. Comparison of Chao1, Shannon, and Simpson indices of **(A)** fecal microbiota, and **(B)** ileal mucosa-associated microbiota (MAM) between MAP-infected and control animals. Statistical analyses were conducted based on PROCMIXED procedure of SAS. The null hypotheses being tested was that the mean of diversity indices of the ileal MAM and the fecal microbiota of infected calves are similar to control group. *P* < 0.05 were considered significant. Trends were discussed at *P* < 0.1.

**Figure 3:**
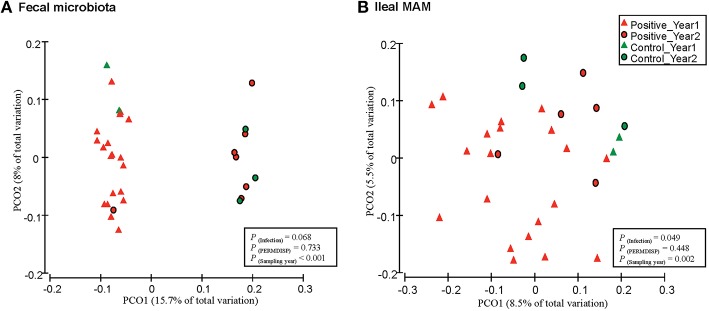
**Principal coordinates analysis (PCoA) of unweighted UniFrac distances**. Between community diversity was tested for impact of MAP infection on clustering patterns of microbiota in **(A)** feces (*P* = 0.068), and **(B)** ileal mucosa (*P* = 0.049). Triangles represent samples belonging to calves that were included in the first year of the infection trial, whereas circles represent samples that belong to the second year. Green and red indicate samples obtained from control and MAP-infected calves, respectively. The impact of MAP infection and sampling year on the clustering pattern of microbial communities were tested using PERMANOVA (implemented in PRIMER-6 software). PERMDIS test was performed to test the null hypothesis of homogeneity of dispersions. *P* < 0.05 were considered significant.

Within community diversity (α-diversity) was calculated using QIIME default scripts. Even depths of 12,000 and 16,000 sequences per sample were used for calculation of Chao1 richness and Shannon and Simpson diversity indices for the ileal MAM and fecal microbiota of MAP-infected and control, respectively (Figure 2). Alpha-diversity indices were also calculated according to the inflammation status (comparison between non-inflamed calves and inflamed ones—calves with minimum score of 1 for both macroscopic lesions and histologic scores), dose or age of infection (Table S1). To compare microbiota composition between samples, β-diversity was measured by calculating phylogenetic-based unweighted UniFrac distances (Lozupone and Knight, 2005). Principal coordinate analysis (PCoA) was applied on resulting distance matrices to generate two-dimensional plots using default settings of the PRIMER-6 (Figure 3; Warwick and Clarke, 2006). Finally, open source software PICRUSt (phylogenetic investigation of communities by reconstruction of unobserved states; Langille et al., 2013) was used to predict functional genes of the classified members of ileal MAM (resulting from reference-based OTU picking against Greengenes database). Predicted genes were then hierarchically clustered and categorized under KEGG (Kanehisa and Goto, 2000) orthologs (KOs) and pathways (level 1–3; Table S2).

### Statistical analyses

The UNIVARIATE procedure of SAS (SAS 9.3, 2012) was used to test the normality of residuals for α-diversity measurements (Table S1). Non-normally distributed data were subjected to box-cox power transformation and then used to assess effects of MAP infection, using MIXED procedure of SAS. The null hypotheses being tested were: (a) the diversity of the ileal MAM and fecal microbiota of MAP-infected calves (based on detection of at least one positive tissue culture) are similar to control group, (b) the diversity of the ileal MAM and fecal microbiota of calves challenged with either high or low doses of MAP at 5 ages are similar; and (c) the diversity of the ileal MAM and fecal microbiota of inflamed calves (all calves with minimum score of 1 for both macroscopic lesions and histologic inflammation) are similar to non-inflamed ones. The effect of block (experimental year) was treated as a random factor in all comparisons. When normality was not achieved, GLIMMIX procedure of SAS was employed to fit Poisson or negative binomial distributions using a log-link function. The goodness-of-fit criteria for each distribution was compared using the −2 log likelihood (smaller is better), Akaike information criterion (AIC; smaller is better), and Pearson's Chi-square over degrees of freedom (DF) ratio (closer to 1 is better). All pairwise comparisons among the groups were tested using Tukey's studentized range adjustment.

Permutational multivariate analysis of variance (PERMANOVA: a FORTRAN computer program for permutational multivariate analysis of variance. Department of Statistics, University of Auckland, New Zealand) implemented in Primer6 software was used to detect significant differences between β-diversity measures of microbial communities. Label permutations were used in PERMANOVA to estimate the distribution of test statistics under the null hypothesis that within-group UniFrac distances are not significantly different from between-group distances (Kuczynski et al., 2010). Infection status (infected vs. control) was considered as a fixed factor whereas the effect of block (experimental year) was treated as random factor in all comparisons. Because PERMANOVA analysis makes the implicit assumption (similar to ANOVA) that dispersions are roughly constant across treatments, PERMDISP test (implemented in Primer-6 software) was performed to check the homogeneity of multivariate dispersions within treatments using phylogenetic-based unweighted UniFrac distances of microbial communities (Figure 3). The PERMDISP test is analogous to PROC UNIVARIATE used above for testing the homogeneity of residuals prior to fitting in ANOVA models.

**Figure 4:**
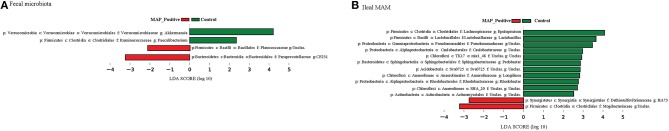
**Phylogenetic comparisons of ileal mucosa-associated (MAM) and fecal microbiota in control and MAP-infected animals**. LEfSe was used to determine differentially abundant genera in **(A)** fecal microbiota, and **(B)** ileal MAM of MAP-infected and control animals. Color code represents the class of treatment (red indicates variables that were significantly enriched in response to MAP infection, whereas green indicates variables that were significantly enriched in the control group).

**Figure 5:**
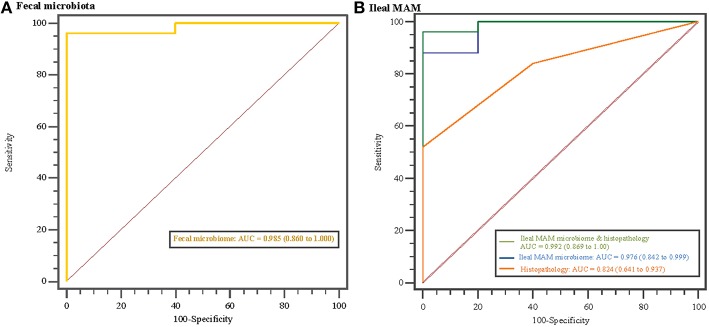
**Disease classification based on microbiome profile and histopathology scores**. Receiver operating characteristics (ROC) curves and area under the curve (AUC) values with 95% confidence intervals in parentheses comparing MAP-infected and control animals based on **(A)** fecal microbiota profile, and **(B)** ileal mucosa-associated microbiota (MAM) profile, histopathology scores, and combination of ileal MAM profile and histopathology scores. The straight line represents the null model.

Statistical analyses regarding relative abundances of bacterial taxa at different phylogenetic levels between MAP-infected and control calves were performed using Linear Discriminant Analysis Effective Size (LEfSe; Segata et al., 2011), software principally developed to discover metagenomics biomarkers. This included the non-parametric factorial Kruskal–Wallis (KW) sum rank test (Kruskal and Wallis, 1952) to test whether the values (the relative abundances of the OTUs summarized at the genus level) in different classes (MAP-infected vs. control) were differentially distributed, then the pairwise Wilcoxon test was used to determine whether all pairwise comparisons between subclasses (experimental years 1 and 2) within different classes agreed with the class level trend. Finally, linear discriminant analysis (LDA) was done to estimate the effect size of each differentially abundant feature. The threshold on the logarithmic LDA score for discriminative features was set at 2.0, so that features with at least 2.0 log-fold changes were considered significant (Figures 4, **7**). The relative abundances of the genera showing the highest LDA scores (revealed by LEfSe analysis) were entered into a logistic regression model and their ability to discriminate group classification was evaluated using area under the receiver operator characteristic (ROC) curve (Figure 5; Delong et al., 1988).

Finally, the relationship between abundant (>1% of community) ileal MAM and histopathological parameters was explored using non-parametric Spearman's rank correlation coefficient test implemented in PAST software (Hammer et al., 2012) and the resulting correlation matrix was visualized in a heatmap format generated by the corrplot package of R (Corrplot: visualization of a correlation matrix. R package version 02-0. 2010. [http://CRAN]). For each correlation, the correlation coefficient (Spearman's ρ) and *P*-value were obtained. The correlation coefficient values ranged from −1 to +1 with larger absolute values indicating stronger relationship while positive and negative values indicating the direction of association.

For all comparisons, statistical significance was considered at a *P* < 0.05. Trends were discussed at *P* < 0.10. *P*-values for fixed effect [*P*_(infection)_], random effect [*P*_(year)_], and homogeneity of multivariate dispersion [*P*_(PERMDISP)_] were calculated and presented separately.

## Results

### Selection of animals based on tissue culture results

As previously reported (Mortier et al., 2013), based on culture of all 21 tissue sites for individual animals, 28 of the 50 MAP-inoculated calves were identified with at least one MAP-positive tissue. In addition, one of the six control calves had MAP-positive tissue sites, and thus was excluded from this study. In total, 28 MAP-positive and five control calves were selected for 16S rRNA profiling of ileal MAM and fecal microbiota (Figure 1).

### Diversity analyses of microbial communities

Illumina paired-end sequencing of ileal MAM and fecal microbiota generated an average of 29,370 and 41,086 high-quality sequences per sample, respectively, with a median sequencing length of 253 nt covering the full length of the V4 hypervariable region of the 16S rRNA gene. At an even depth of 16,000 and 12,000 sequences per sample, clustering of OTUs at 97% similarity threshold resulted in Good's non-parametric coverage estimates (Good, 1953) of 97.35 and 99.45% for fecal and ileal MAM, respectively. In general, comparing the Chao1 richness and Shannon and Simpson diversity indices within each sampling site revealed no apparent significant differences between MAP-infected and control animals although some trends were observed (Figure 2). Similar results were observed with regards to inflammation status (comparison between non-inflamed calves and inflamed ones—calves with minimum score of 1 for both macroscopic lesions and histologic scores), dose or age of infection (Table S1).

Furthermore, PERMANOVA analyses of unweighted UniFrac distances revealed that fecal microbiota of MAP-infected animals tended to cluster separate from control group [*P*_(infection)_ = 0.068 and *P*_(PERMDISP)_ = 0.733; Figure 3A]. In addition, there was a distinct clustering pattern between ileal MAM [*P*_(infection)_ = 0.049 and *P*_(PERMDISP)_ = 0.448] of infected and control animals (Figure 3B). For both sampling sites, the impact of block (sampling year) on composition of bacterial communities was significant [*P*_(year)_ < 0.001; Figures 3A,B].

### Taxonomic classification of 16S rRNA gene sequences

Alignment of OTUs at 97% similarity threshold against Greengenes database resulted in identification of 29 and 14 bacterial phyla, and 489 and 244 bacterial genera within the ileal MAM and fecal microbiota, respectively. While majority of OTUs were identified at genus (g.) or species levels, some were only classified at the phylum (p.), class (c.), order (o.), or family (f.). Proportions of the abundant (>1% of community) bacterial phyla and genera have been presented in Table 1. Proportions of the most abundant representative OTUs (>1% of community) in MAP-infected vs. control calves, regardless of significance, were depicted in Figure S1.

**Table 1:** **Relative abundances of abundant (>1% of community) bacterial phyla and genera in control and MAP-infected samples**.

**Phylum**	**Genus**	**Proportion (%)** ± ***SD***^a^	
		**Control**	**MAP-positive**
**FECAL MICROBIOTA**			
Bacteroidetes		11.10 ± 5.20	16.81 ± 6.11
	Uncl. Bacteroidales	1.38 ± 0.94	4.89 ± 3.84
	Uncl. Bacteroidaceae	0.76 ± 0.61	3.40 ± 4.23
	Uncl. Rikenellaceae	3.49 ± 3.72	1.63 ± 1.43
	Uncl. S24-7	0.92 ± 1.06	1.67 ± 1.25
	5-7N15	3.45 ± 2.04	3.31 ± 1.79
Firmicutes		80.25 ± 3.27	78.03 ± 6.19
	Clostridium	1.96 ± 0.63	2.04 ± 0.42
	Uncl. Clostridiaceae	9.38 ± 4.52	6.96 ± 3.11
	Uncl. Clostridiales	15.93 ± 3.24	14.53 ± 2.70
	Dorea	1.69 ± 2.30	0.96 ± 0.45
	Uncl. Lachnospiraceae	2.33 ± 1.17	2.41 ± 0.93
	Uncl. Mogibacteriaceae	1.26 ± 0.67	1.12 ± 0.34
	Uncl. Peptostreptococcaceae	10.86 ± 6.77	8.26 ± 4.04
	Ruminococcus	1.41 ± 0.84	1.33 ± 0.64
	Uncl. Ruminococcaceae	27.71 ± 7.38	32.93 ± 7.46
	SMB53	1.11 ± 0.43	0.87 ± 0.37
	Turicibacter	1.40 ± 1.19	1.86 ± 1.17
Proteobacteria		1.79 ± 3.74	0.34 ± 0.40
	Sphingomonas	1.63 ± 3.64	0
Tenericutes		1.98 ± 1.02	2.99 ± 1.01
	Uncl. RF39	1.82 ± 0.97	2.68 ± 1.02
Verrucomicrobia		3.56 ± 4.04	0.57 ± 0.71
	Akkermansia	3.55 ± 4.05	0.57 ± 0.71
**ILEAL MUCOSA-ASSOCIATED MICROBIOTA**			
Actinobacteria		2.27 ± 0.81	3.38 ± 4.85
	Bifidobacterium	0.85 ± 0.84	1.00 ± 0.95
Bacteroidetes		16.73 ± 8.51	14.55 ± 736
	Bacteroides	3.51 ± 1.81	3.07 ± 1.60
	Uncl. Bacteroidales	3.96 ± 2.23	3.05 ± 1.84
	Porphyromonadaceae	2.00 ± 1.06	1.50 ± 0.95
	Prevotella	3.82 ± 2.31	3.61 ± 2.15
	Uncl. S24-7	1.26 ± 0.71	1.15 ± 0.78
Chloroflexi		1.48 ± 0.94	0.79 ± 0.52
Cyanobacteria		1.67 ± 2.60	0.70 ± 0.85
	Uncl. Streptophyta	1.13 ± 2.53	0.14 ± 0.60
Firmicutes		56.33 ± 12.34	64.96 ± 14.16
	Allobaculum	1.44 ± 0.76	1.09 ± 0.67
	Blautia	2.97 ± 2.40	1.98 ± 1.11
	Butyrivibrio	1.46 ± 1.13	1.10 ± 0.78
	Clostridium	2.30 ± 1.19	2.91 ± 3.27
	Uncl. Clostridiales	6.57 ± 1.02	9.46 ± 4.27
	Uncl. Clostridiaceae	8.30 ± 7.91	9.76 ± 6.01
	Coprococcus	2.27 ± 1.49	1.49 ± 0.98
	Epulopiscium	5.67 ± 1.28	3.27 ± 1.82
	Lactobacillus	1.74 ± 0.85	0.82 ± 0.45
	Uncl. Lachnospiraceae	2.28 ± 1.19	1.92 ± 1.08
	Uncl. Peptostreptococcaceae	8.76 ± 9.81	13.99 ± 9.90
	Uncl. Ruminococcaceae	3.49 ± 1.13	4.76 ± 3.16
	Ruminococcus	2.67 ± 1.32	2.52 ± 1.01
	Turicibacter	1.26 ± 1.87	2.44 ± 2.33
Proteobacteria		17.10 ± 3.59	10.41 ± 5.63
	Campylobacter	1.17 ± 0.77	0.68 ± 0.52
	Uncl. Helicobacteraceae	1.99 ± 1.26	1.29 ± 1.09
	Pseudomonas	4.30 ± 2.41	3.20 ± 2.09
	Uncl. Xanthomonadaceae	1.03 ± 0.53	0.57 ± 0.38
Tenericutes		0.79 ± 0.45	2.46 ± 2.10
	Uncl. RF39	0.69 ± 0.45	2.30 ± 2.10

a *SD: Standard deviation of the mean*.

### Utilizing fecal and ileal MAM microbiota profile for distinguishing between MAP-infected and control animals

Overrepresentation of bacterial taxa—at different taxonomic levels—in response to MAP-infection were further characterized using LEfSe analysis (Figure 4). In fecal microbiota, the proportion of members of the p. Verrucomicrobia (including g. *Akkermansia*), and p. Firmicutes (including g. *Faecalibacterium*) were higher in control calves, whereas the proportion of the unclassified Planococcaceae (p. Firmicutes) and g. *CF231* (p. Bacteroidetes) enriched in MAP-infected calves (Figure 4A and Figure S2). The relative abundances of the OTUs aligned to these four bacterial genera significantly differentiated the fecal microbiota of MAP-infected and control animals (AUC = 0.985, *P* < 0.001, Figure 5A). Conversely, several members of the p. Proteobacteria were relatively more abundant in the ileal MAM of controls compared to MAP-infected calves (Figure 4B). These included members of the c. Alphaproteobacteria (including f. Sphingomonadaceae), c. Gammaproteobacteria (including unclassified members of f. Pseudomonadaceae). Within the phylum Firmicutes, g. *Lactobacillus* and *Epulopiscium* were overrepresented in the ileum of control calves, whereas the proportion of the unclassified members of the f. Mogibacteriaceae (p. Firmicutes) were increased in MAP-infected calves. The relative abundances of the OTUs aligned to *Lactobacillus, Epulopiscium*, unclassified members of Pseudomonadaceae, and Mogibacteriaceae significantly differentiated the MAP-infected and control animals (AUC = 0.976, *P* < 0.001, Figure 5B). In addition, a combination of ileal MAM microbiota profile and histopathology scores (macroscopic and histological lesions) showed increased discriminative power over the use of microbiota profile alone (AUC = 0.992, *P* < 0.001, Figure 5B).

### Correlation between mucosa-associated microbiota and histopathology parameters

**Figure 6:**
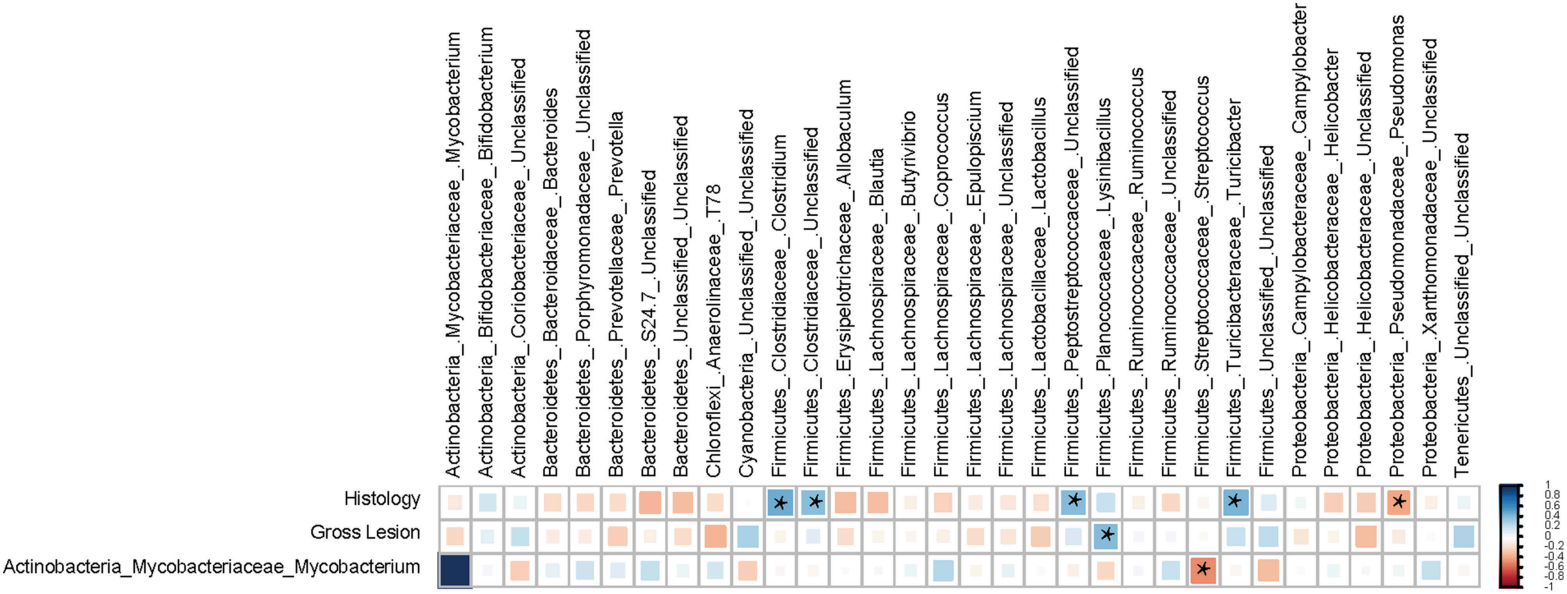
**Spearman's correlation coefficient analysis**. The correlation matrix was based on the relationship of the abundant ileal mucosa-associated bacterial genera (>0.1% of community) with the severity of macroscopic and histological lesions. The strength of the correlation between each pair of variables is indicated by the size and color intensity of the squares. A color code of dark blue indicates a positive correlation coefficient close to +1, whereas dark red indicates a negative correlation coefficient close to –1. All pairwise correlations with a *P* < 0.05 were considered significant and indicated by “^*^”. The last row of the matrix was included to explore the correlation between the proportion of genus *Mycobacterium* and other abundant members of the mucosa-associated microbiota (MAM).

There were significant correlations between the ileal MAM and occurrence of macroscopic and histological lesions. Within the p. Firmicutes, members of the g. *Clostridium* (Spearman's ρ = +0.48 and *P* = 0.008) and *Turicibacter* (Spearman's ρ = +0.46 and *P* = 0.012), and f. Peptostreptococcaceae (Spearman's ρ = +0.42 and *P* < 0.022) had strong positive correlations with severity of histological lesions, whereas g. *Pseudomonas* (within p. Proteobacteria) were negatively correlated (Spearman's ρ = −0.38 and *P* = 0.041) with severity. The g. *Lysinibacillus* (p. Firmicutes) were the only member of the ileal MAM positively correlated (Spearman's ρ = +0.42 and *P* = 0.023) with occurrence and severity of macroscopic lesions. Exploring co-occurrence of the g. *Mycobacterium* with other abundant members of the ileal MAM also suggested a strong negative correlation between members of this genus and those of the g. *Streptococcus* (p. Firmicutes; Figure 6).

### Alterations in functional properties of microbiota in response to MAP-infection

**Figure 7:**
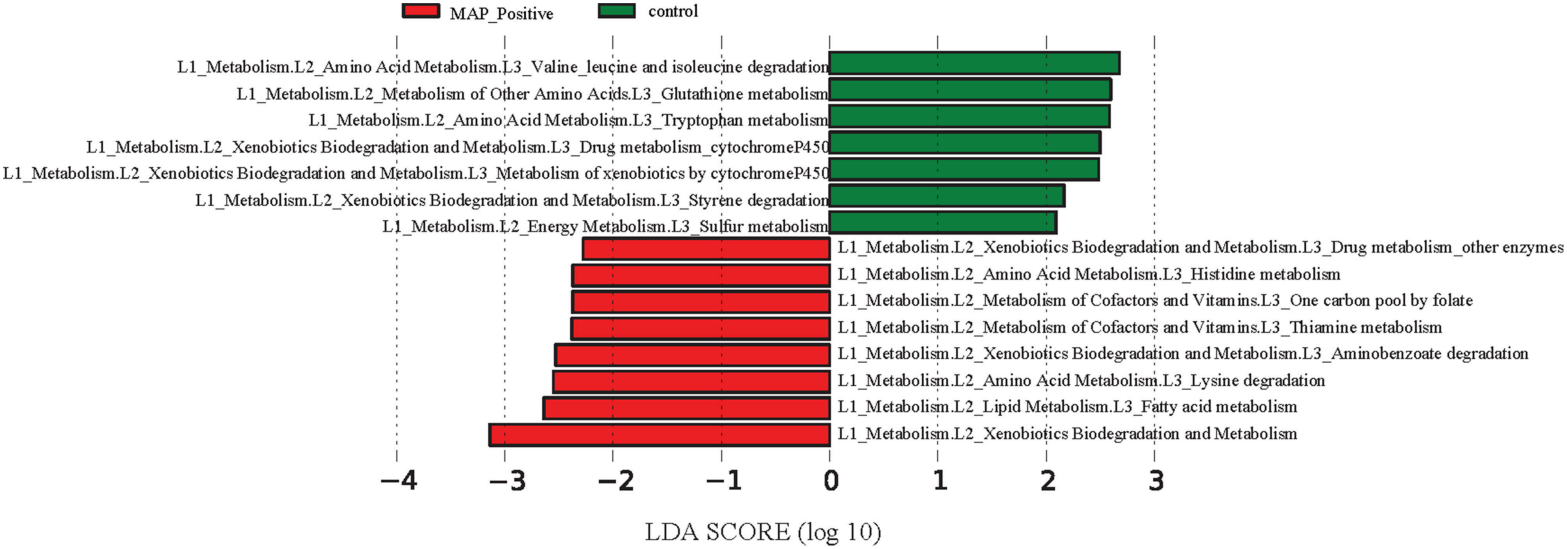
**Differentiation of functional metagenomes of ileal mucosa (MAM) and fecal microbiota in control and MAP-infected calves**. A Statistical differences between KEGG pathways (explored at Levels 1–3, indicated as L1–L3) of ileal MAM metagenomes were evaluated by LEfSe, a metagenome analysis approach which performed the linear discriminant analysis following the Wilcoxon Mann–Whitney test to assess effect size of each differentially abundant variable. The length of the horizontal bars indicated log-fold changes for each variable. Color code represents the class of treatment (red indicates variables that were significantly altered in response to MAP infection, whereas green indicates variables that were significantly associated with the control group).

The reference-based OTU picking resulted in 80.03% of the sequencing reads being mapped to the Greengenes database. Consequently, a high proportion of MAM were utilized for metagenomic predictions. Major KEGG pathways including valine, leucine, and isoleucine degradation, as well as glutathione and tryptophan metabolism exhibited more than 2.5 log-fold increase within the ileal MAM of control calves, whereas the proportion of fatty acid, lysine, thiamine, and histidine metabolism pathways were enriched in the ileal MAM of the MAP-infected group (Figure 7).

## Discussion

The present study was the first attempt to characterize microbial dysbiosis following MAP-induced GI inflammation. Notwithstanding, characteristics of gut microbiota dysbiosis, including general pattern and indicator taxa, have been investigated as potential biomarkers for detection/prediction of chronic gut inflammatory diseases (Berry and Reinisch, 2013). Exploring global shifts in the composition of the ileal MAM and fecal microbiota of infected cattle identified several microbial fingerprints with potential applications for tracking JD during subclinical asymptomatic stages. Additionally, the reconstructed metagenomics approach (PICRUSt) used in the current study revealed changes in the functional properties of the ileal MAM after MAP infection, suggesting potential roles for this dynamic network of microorganisms in pathogenesis of intestinal inflammation.

Experimentally infected calves in this study were exposed to either high or low doses of viable MAP, which corresponds to the range of doses that calves would be exposed to under natural conditions on farm. Therefore, we inferred that the outcomes were more relevant for clinical use. Although the success rate for experimental induction of MAP infection varies greatly and results are prone to errors produced by low sensitivity of current tests (Hines et al., 2007), the combination of methods used for identification of MAP-positive calves [positive interferon-gamma release assay (Mortier et al., 2014c), fecal shedding (Mortier et al., 2014b), antibody ELISA (Mortier et al., 2014a), and MAP culture of intestinal tissues along with gross and histopathological lesion scoring (Mortier et al., 2013)] demonstrated a high success rate of exposure and infection in these inoculated calves. However, inclusion criteria for defining MAP-infected and control calves for the purpose of this study, were based on stringent criteria of presence and absence of tissue-culture positive sites, commonly accepted as the gold standard for identification of MAP-infected animals (Tiwari et al., 2006).

The ileal MAM and fecal microbiota of inflamed and non-inflamed calves (based on macroscopic or histologic inflammatory responses) did not differ in richness and biodiversity of microbial communities (Table S1). Although, this trend has not always been consistent (Lepage et al., 2005), an overall decrease of microbial diversity and stability has been generally reported in subjects with chronic GI inflammation (Willing et al., 2010; Michail et al., 2012). The majority of these studies were based on the comparison between control subjects with those at clinical stages of IBD; therefore, we speculate that the unchanged biodiversity of intestinal microbiota of MAP-infected calves in the current study (Figure 2) was due to a lack of severe inflammatory responses during subclinical stages of JD.

Comparison of unweighted UniFrac distances of microbial communities revealed distinct clustering patterns of the ileal MAM and fecal microbiota in MAP-infected vs. control calves (Figure 3). The effect of sampling year was the most discriminative factor in clustering patterns of both mucosa and fecal microbial communities; most likely this was due to differences in ambient temperature at sampling (summer vs. winter in Years 1 and 2, respectively) or dietary factors that impacted microbiota composition. By considering experimental year as a random factor in PERMANOVA analysis, impacts of infection on the composition of the ileal MAM and fecal microbiota were significant.

Previous studies have reported the occurrence of the so-called “microbial dysbiosis” in feces (Joossens et al., 2011) and intestinal mucosa (Walker et al., 2011) of IBD patients, which can co-occur with gastrointestinal inflammation and result in compromised interaction between the host immune system and microbiota (Hooper et al., 2012). Colonization and replication of MAP within intestinal mucosa, considered the first sign of infection, can occur as early as 1 month post exposure (Whittington and Sergeant, 2001). As reported (Mortier et al., 2013), in the current study, macroscopic and histologic lesions were detected in 62 and 84% of inoculated animals, respectively. In a parallel study to this work, evaluation of transcripts in whole blood samples revealed that at 3 months post infection, IL-6 signal transducer (ST) gene was upregulated in the MAP-infected calves compared to the control group (David et al., 2014). The IL6ST/gp 130 is known to regulate transduction of proinflammatory cytokines, i.e., IL-6 and IL-27 (Silver and Hunter, 2010), which their increased production has been reported in CD patients with chronic colitis (Yamamoto et al., 2000; Lovato et al., 2003). These data together suggest development of a chronic inflammation in the GI tract of infected calves, which could have promoted microbial dysbiosis. The presence of dysbiosis was further confirmed by obtaining high AUC values (>0.97; Figure 5B) when using a combination of microbiome profile and histopathology scores, or microbiome profile alone, to differentiate between MAP-infected and control calves. The logistic model used for this purpose included OTUs belonging to genera *Lactobacillus* and *Epulopiscium* (overrepresented in the ileal MAM of control calves), as well as unclassified members of the f. Mogibacteriaceae (enriched in the ileal MAM of MAP-infected calves); unraveling their contribution in development of MAP-specific microbial dysbiosis.

There were positive correlations between members of g. *Clostridium* and f. Peptostreptococcaceae with histopathology scores, whereas g. *Pseudomonas* was negatively correlated to tissue inflammation (Figure 6). Although the role of f. Peptostreptococcaceae and g. *Pseudomonas* in development of mucosal inflammation is poorly understood, commensal species within the g. *Clostridium* can modulate inflammatory responses in intestinal mucosa by stimulating expression of T_reg_ cells (Atarashi et al., 2011). The positive correlation of g. *Clostridium* with histopathology scores in the current study might have be either due to the specific characteristics of microbiota-host immune interaction in ruminant, or more likely due to the lack of resolution of 16S rRNA gene sequencing methodology in differentiating several strains within this bacterial lineage. *Clostridium difficile* is one of the pathogens belonging to this genus that produces inflammatory enterotoxins (Pothoulakis and Lamont, 2001) and has been associated with IBD (Rodemann et al., 2007). Therefore, care should be taken when making inferences about disease pathogenesis based on compositional changes of microbiota based on 16S rRNA profiling.

Based on predicted metagenomes (PICRUSt) of the ileal MAM, we inferred that there were several functional pathways associated with MAP infection (Figure 7). An interesting characteristic of inflammation-associated dysbiosis is the ability of certain bacteria to utilize host substrates to gain a fitness advantage during inflammation (Rooks et al., 2014). In the current study, genes encoding amino acid metabolism pathways of histidine and lysine were more abundant in the MAM of MAP-infected calves, whereas concurrently, there was an underrepresentation of degradation pathways for valine, leucine, and isoleucine, as well as glutathione and tryptophan metabolism pathways. Biosynthesis of glutathione, by members of p. Proteobacteria and few streptococci and enterococci strains, contributes to maintain bacterial homeostasis during oxidative stress caused by inflammation (Sherrill and Fahey, 1998; Morgan et al., 2012). Furthermore, underrepresentation of glutathione metabolism pathways of MAP-infected calves corresponded well to the decreased proportion of the members of p. Proteobacteria in the ileal MAM of infected animals. This could have been due to unfavorable growth conditions in inflamed mucus for this bacteria lineage, which, in turn, might have resulted in impaired ability of microbiota of infected calves to maintain homeostasis. Lending support to the abovementioned findings, De Buck et al. (2014) in a parallel study to this experiment reported increased concentrations of branched chain amino acids leucine and isoleucine in the serum of the MAP-infected calves. Likewise, enrichment of histidine metabolism pathways in the ileal MAM of infected calves also corresponded to the increased serum concentrations of this amino acid in the same group of calves (De Buck et al., 2014). Collectively, these results suggest that dysbiosis is not simply limited to compositional changes in intestinal microbiota, but rather it includes major impairments in fundamental microbial metabolic functions that can affect the physiology of host animal and alters its metabolomic profile.

Since the composition and diversity of small intestine microbiota differs from fecal microbiota (Marteau et al., 2001; Lepage et al., 2005) and the application of the latter, due to non-invasive sample procurement, is preferred for biomarker discovery, characteristics of MAP-associated dysbiosis of fecal microbiota were further investigated (Figures 4A, 5A). The combined logistic model used for distinguishing the fecal microbiota of MAP-infected animals from the control group was based on the relative abundances of the OTUs that are aligned to f. Planococcaceae and Paraprevotellaceae (overrepresented in MAP-infected fecal microbiota), as well as g. *Faecalibacterium* and *Akkermansia* (underrepresented in MAP-infected fecal microbiota). Fitting the collection of these OTUs in the logistic model resulted in high AUC value (0.985), suggesting their potential to serve as biomarker for identifying infected cattle during subclinical stages of JD. Underrepresentation of g. *Akkermansia* and *Faecalibacterium* in the fecal microbiota of infected calves was in agreement with the trend seen in IBD patients (Berry and Reinisch, 2013). Members of the g. *Faecalibacterium* are sensitive to inflammatory changes in intestinal ecosystem (Berry and Reinisch, 2013), most likely due to changes in concentration of metabolites (i.e., increased bile acid concentration) or increased reactive oxygen stress (Jansson et al., 2009; Morgan et al., 2012), which could make them ideal biomarkers of GI inflammation. Moreover, underrepresentation of the g. *Akkermansia* in fecal microbiota of MAP-infected calves could also be indicative of intestinal inflammation. Following chronic inflammation, the overall abundance of mucolytic bacteria usually increases in the mucus layer of the IBD patients; however, inflammatory-associated changes in mucin composition predispose to overgrowth of specific mucolytic species within g. *Ruminococcus* (Png et al., 2010; Berry and Reinisch, 2013). Consequently, the proportion of the g. *Akkermansia*, a dominant and native mucin degrader of the intestinal mucosa, has been reported to be higher in healthy subjects (Berry and Reinisch, 2013).

## Conclusions

Our metagenomics approach identified microbes and microbial functions associated with MAP infection. Based on compositional and predicted functional shifts in the ileal MAM, we inferred that this dynamic network of microorganisms has a potential role in development of intestinal inflammation following MAP infection. Furthermore, microbiota composition of the feces of infected calves had promised as biomarker candidates for differential diagnosis of JD during subclinical stages of the disease. Simultaneous overrepresentation of f. Planococcaceae and Paraprevotellaceae and underrepresentation of g. *Faecalibacterium* and *Akkermansia* in the fecal microbiota of infected cattle could serve as indicators of MAP infection. Notwithstanding, the sensitivity and specificity of these markers to detect MAP infection under field conditions need to be validated in large-scale field studies.

## Author contributions

JD, HB, DK conceived and designed the experiment. RM conducted the animal experiment. HD performed lab analyses. HD and EK developed the bioinformatics and statistical models and analyzed the data. HD, JD, HB, and EK wrote the manuscript.

## Funding

The research was funded by Manitoba Rural Adaptation Council (MRAC) of Canadian Agricultural Adaptation Program (CAAP) and Manitoba Cattle Producer Association (MCPA). The animal experiment was supported by Dairy Farmers of Canada (PESAC program), Natural Science and Engineering Research Council of Canada Collaborative Research and Development Program (NSERC-CRD), Alberta Milk, and Alberta Livestock and Meat Agency.

### Conflict of interest statement

The authors declare that the research was conducted in the absence of any commercial or financial relationships that could be construed as a potential conflict of interest.
