# Supplementary material for: The Features of Fecal and Ileal Mucosa-Associated Microbiota in Dairy Calves during Early Infection with Mycobacterium avium Subspecies paratuberculosis
Source: Front Microbiol. 2016 Mar 31;7:426. doi: 10.3389/fmicb.2016.00426 (PMC4814471; doi:10.3389/fmicb.2016.00426)
Supplement: Supplementary file 1 [file Table1.PDF]

Table S1. Summary statistics for diversity indices observed in microbial communities of fecal and mucosa-associated microbial communities

Diversity index	Mean values for indicated indices									Test statistics							
	Dose[1]		Age[2]					Inflammation[3]		Dose		Age		Dose× Age		Inflammation	
	High (n=14)	Low (n=14)	2 wk (n=4)	3 mo (n=6)	6 mo (n=5)	9 mo (n=6)	12 mo (n=7)	Non-inflamed (n=18)	Inflamed (n=15)	SED[4]	*P*-value	SED	*P*-value	SED	*P*-value	SED	*P*-value
---Feces---																	
**Chao1**	1358.08	1355.43	1366.31	1351.68	1333.46	1394.45	1337.89	1347.14	1355.48	27.417	0.92	42.928	0.56	60.228	0.08	25.866	0.74
**Observed Species**	1083.26	1090.40	1092.08	1085.23	1055.74	1119.85	1081.27	1081.36	1094.69	18.014	0.69	28.184	0.33	39.560	0.16	17.843	0.46
**Shannon**	7.844	7.770	7.641	7.943	7.608	8.079	7.765	7.855	7.763	0.118	0.53	0.185	0.10	0.259	0.31	0.120	0.44
**Simpson**	0.983	0.981	0.977^{a}	0.986^{ab}	0.977^{a}	0.989^{b}	0.982^{ab}	0.984	0.983	0.002	0.35	0.003	0.01	0.005	0.08	0.003	0.66*
---Ileal mucosa---																	
**Chao1**	529.62	526.93	648.78	456.96	540.87	488.85	505.91	533.16	480.44	42.963	0.95	67.994	0.16	95.366	0.95	38.814	0.18
**Observed Species**	461.47	458.18	567.97	409.79	443.96	435.94	441.46	473.38	418.35	37.665	0.93	59.496	0.22	83.433	0.98	31.782	0.09
**Shannon**	6.466	6.828	7.143	6.774	5.624	6.802	6.891	6.801	6.697	0.295	0.24	0.466	0.09	0.653	0.46	0.251	0.68*
**Simpson**	0.958	0.968	0.978	0.967	0.933	0.962	0.974	0.970	0.965	0.012	0.38	0.019	0.27	0.028	0.91	0.008	0.54*

[a-c] Within a row, means without a common superscript differed ($P < 0.05$).
[*]Statistical analyses were conducted based on GLIMMIX procedure of SAS.
[1]Dose of inoculum used for MAP infection challenge: high (5×10^{9} CFU) or low (5×10^{7} CFU).
[2]Age at which calves were inoculated with MAP (all animals were euthanized at 17 month of age).
[3]Comparison between inflamed (all animals with minimum score of 1 for macroscopic lesions and histology) and non-inflamed calves.
[4]SED = standard error of difference between treatment means.
