# Supplementary figures and images for: The Features of Fecal and Ileal Mucosa-Associated Microbiota in Dairy Calves during Early Infection with Mycobacterium avium Subspecies paratuberculosis
Source: Front Microbiol. 2016 Mar 31;7:426. doi: 10.3389/fmicb.2016.00426 (PMC4814471; doi:10.3389/fmicb.2016.00426)

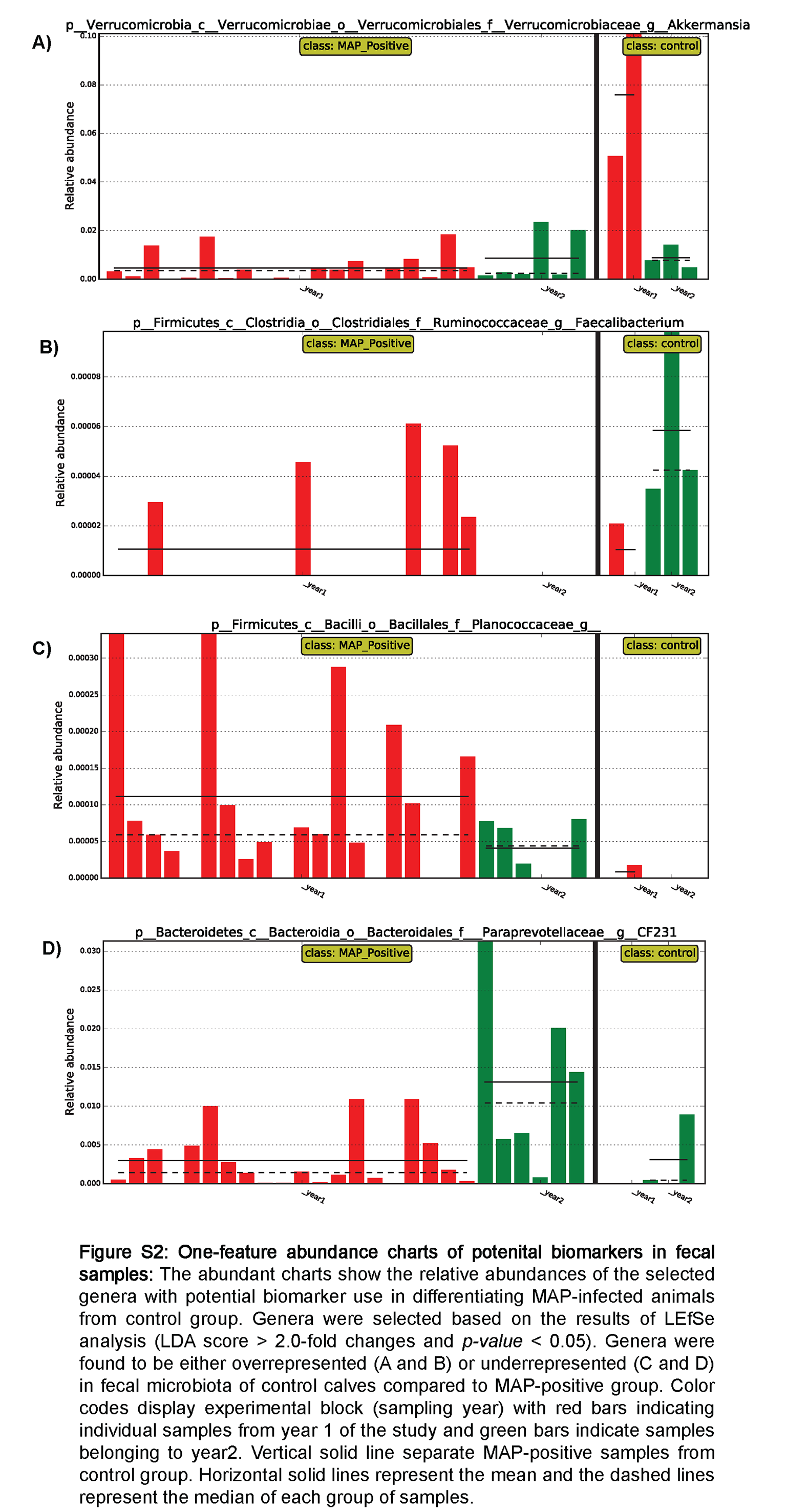

Supplement: Supplementary file 3 [file Image1.TIFF]

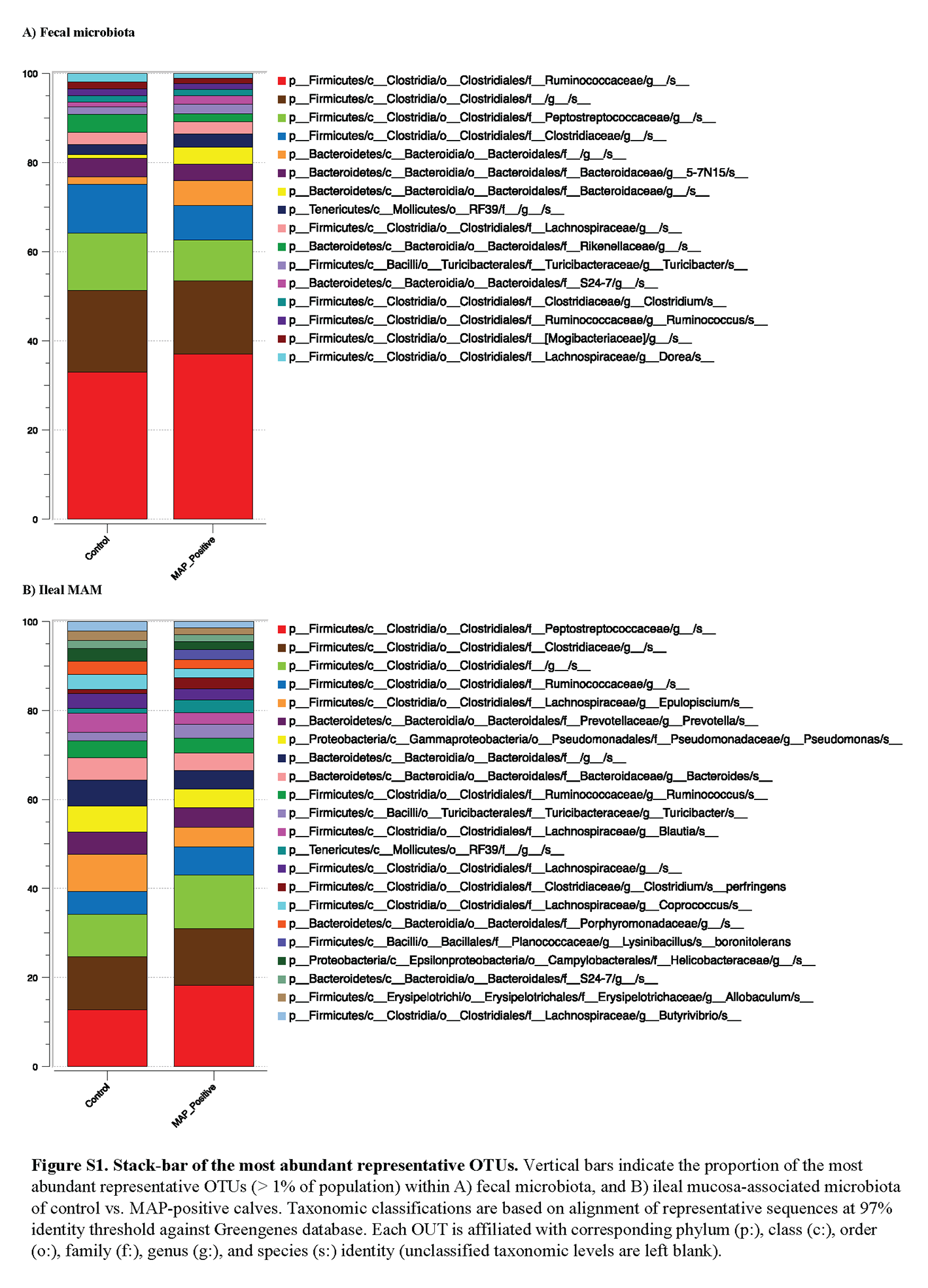

Supplement: Supplementary file 4 [file Image2.TIFF]
